# Effects of interdisciplinary teamwork on patient-reported experience of cancer care

**DOI:** 10.1186/s12913-017-2166-7

**Published:** 2017-03-20

**Authors:** Dominique Tremblay, Danièle Roberge, Nassera Touati, Elizabeth Maunsell, Djamal Berbiche

**Affiliations:** 1 Nursing School, Faculty of Medicine and Health Sciences, Université de Sherbrooke, Longueuil, Quebec Canada; 2 Charles-Le Moyne Hospital Research Center, Greenfield Park, Quebec Canada; 3 Department of Community Health Sciences, Faculty of Medicine and Health Sciences, Université de Sherbrooke (Longueuil Campus), Longueuil, Quebec Canada; 4 École nationale d’administration publique, Montreal, Quebec Canada; 5 Department of Social and Preventive Medicine, Faculty of Medicine, Université Laval, Québec City, Quebec Canada

**Keywords:** Cancer care, Interdisciplinary teamwork, Patient care team, Patient-reported experience measures, Quasi-experimental study

## Abstract

**Background:**

Interdisciplinary teamwork (ITW) is deemed necessary for quality cancer care practices. Nevertheless, variation in ITW intensity among cancer teams is understudied, and quantitative evidence of the effect of different ITW intensities among cancer teams on patients’ perceived experience of care is limited. This study aims to compare patient-reported experience measures (PREMs) of cancer outpatients followed by teams characterized by high *vs.* low ITW intensity.

**Methods:**

The study is designed as an *ex post facto* quasi-experimental study. Participants (*n* = 1379) were recruited in nine outpatient oncology clinics characterized by different ITW intensities. ITW intensities were evaluated using the characteristics of structure (team composition and size) and process (interactions among team members), as per West’s seminal work on team effectiveness. ITW intensity was dichotomized (high *vs.* low ITW intensity). PREMs were classified and measured using validated scales corresponding to six dimensions: *Prompt access to care*, *Person-centred response*, *Quality of patient-professional communication*, *Quality of the care environment*, *Continuity of care*, and *Results of care*. Dichotomous variables were created for each dimension (positive *vs.* less positive experience). Multiple logistic regression analyses were performed to assess the association between ITW intensities and the six PREMs dimensions, while controlling for patient and organizational characteristics. PROC GENMOD was used to fit logistic models for categorical variables.

**Results:**

Outpatients treated by teams characterized by high ITW intensity reported almost four times more positive perceptions of *Prompt access to care* compared to patients treated by low ITW intensity teams (OR = 3.99; CI = 1.89–8.41). High ITW intensity also positively affected patients’ perceptions of *Quality of patient-professional communication* (OR = 2.37; CI = 1.25–4.51), *Person-centred response* (OR = 2.11; CI = 1.05–4.24], and *Continuity of care* (OR = 2.18; CI = 1.07–4.45). No significant association was found between ITW intensity and perceived *Results of care* (OR = 1.31; CI = 0.68–2.52) or *Quality of the care environment* (OR = 0.66; CI = 0.31–1.39).

**Conclusions:**

This study provides empirical evidence, from the patient’s perspective, that ITW intensity affects some critical aspects of patient-reported quality of care. Future research will allow explaining how and why ITW structure and processes may contribute to positive cancer care experiences.

**Electronic supplementary material:**

The online version of this article (doi:10.1186/s12913-017-2166-7) contains supplementary material, which is available to authorized users.

## Background

Interdisciplinary teamwork (ITW) is recognized as a gold standard for the management of cancer patients and is promoted by leading organizations such as the European Partnership for Action Against Cancer (EPAAC) [1] and the American Society of Clinical Oncology (ASCO) [2]. Given the multiple potential benefits and the goal of providing all patients with comprehensive care, ITW represents both a rational and ethical approach to care. ITW involves health care professionals working as a team with the purpose of discussing individual cases and recommending care plans. Teamwork has been defined in several ways. The definition of ITW used in this article involves an alliance of all medical and health care professionals related to a specific tumor site. Their approach to cancer care is guided by their willingness to agree on evidence-based clinical decisions and to deliver coordinated care throughout the cancer care continuum, while patients are encouraged to take an active role in their care [1]. This definition relates to specific aspects of cancer care and is similar to those used in the general health care sector [3, 4]. Typically, interdisciplinary cancer care teams include clinicians specialized in oncology, pathology, pharmacology, and psychosocial and nursing care in an oncology setting. Other relevant professionals with training that is not specific to oncology may also be part of the cancer care team [5].

Recent systematic reviews have supported the rationale for ITW as a way to improve cancer patient outcomes and survival, notably with respect to clinical benefits based on personalized health care decisions [5–7]. Despite these encouraging observations, in-depth appraisal of the quality of evidence raises concerns about inferring a direct causal relationship between ITW and patient experience [8]. First, the description of health care teams has been incomplete and, as a result, the operationalization of ITW concepts and measures has been inconsistent [7, 9]. Second, the heterogeneity of interventions considered as ITW (including “comprehensive cancer team,” “tumor board,” and “multidisciplinary cancer conference”), and outcomes used to assess the effects of ITW, makes it impossible to generalize the results or identify ITW key elements that affect patient-reported experience measures [10]. For example, cancer care resulting from ITW focusing on pathology may produce different effects than ITW focusing on psychosocial aspects, quality of life, patient empowerment, and patient rights. Third, although the most effective way to test the effects of ITW would be to conduct a large, parallel-group, randomized controlled trial (RCT), such a research design is impossible given that ITW has already been implemented to varying degrees and that its implementation is critically dependent on context [11]. Moreover, an RCT could not be validly conducted given that most cancer team members naturally work together to face the complexity of cancer care. Finally, most studies aimed at determining the effect of teamwork have focused on patient outcomes or patient satisfaction and not on PREMs dimensions. While this type of evaluation provides useful insight into patients’ perception of services against their expectations, it offers little information on the actual processes at play during active care. These observations indicate uncertainty about the association between ITW and patient perception of their care experience [12, 13]. Such evidence nonetheless suggests the following hypothesis: the greater the intensity of ITW in cancer care teams, the more extensive the beneficial effects perceived by patients. This study aims to compare ITW intensity with PREMs dimensions of care among cancer outpatients.

### Conceptual framework

**Fig. 1:**
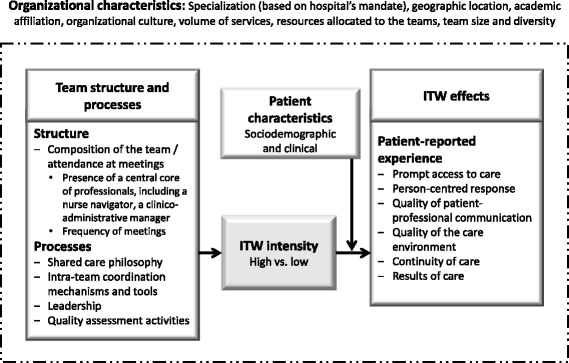
Conceptual framework for interdisciplinary teamwork (ITW) in cancer team. Adapted from Tremblay [12]

Our group developed a conceptual framework [12] that was adapted to guide the study. We constructed our framework (Fig. 1) based on a review of the relevant scientific and grey literature to ensure systematic identification of the most critical ITW indicators. It was constructed using commonly accepted structure-process-outcome associations, building on the work of West et al. [14–16]. The framework illustrates the convergence of structure and process characteristics that affect ITW intensity. Structure characteristics include composition of the team (suggested by both the scientific literature and the Quebec cancer program); attendance at team meetings by team members (central professional core, nurse navigator, clinical-administrative manager); and frequency of meetings. Process characteristics include shared philosophy of care (extent to which values, goals, and objectives are shared by team members); intra-team coordination mechanisms and tools (presence of a cancer care coordinator, also known as oncology pivot nurse or nurse navigator/coordinator); leadership (involving both the physician and front-line manager); and quality assessment activities (including continuing education opportunities for gaining new knowledge and sharing expertise, and systems for capturing patient satisfaction). In this study, it is hypothesized that high ITW intensity is associated with PREMs corresponding to positive patient perception of the following dimensions of cancer care: *Prompt access to care*, *Person-centred response*, *Quality of patient-professional communication*, *Quality of the care environment* (basic amenities and professional courtesy), *Continuity of care*, and *Results of care*. The framework also shows that the characteristics of cancer patients and the organizational context of cancer teams may affect ITW intensity and thus ultimately the patient’s perceived experience. Similarly, the characteristics of cancer patients may influence their perception of the dimensions of care.

### Study context

The study was conducted in the Province of Quebec, Canada. The province has a publicly funded health care system providing universal access to medical services for over eight million residents. Quebec’s initial cancer control plan was launched in 1998 to enhance the accessibility, coordination, continuity, and responsiveness of patient-centred care [17]. Among other things, the plan prioritized service reorganization by defining and designating local, regional, and supra-regional mandates for all hospital cancer teams. Regional and supra-regional mandates are differentiated in terms of their degree of specialization of cancer services, the radiotherapy they provide, and their consulting role. The plan provides explicit guidelines for team structure and processes and proposes a number of initiatives to strengthen the functioning of all local cancer teams (*n* = 65). These include continuing education programs for inter-professional collaboration, a description of care coordinator tasks and responsibilities, and an accreditation process for cancer teams. Quebec’s cancer control plan defines local teams as a core of professionals including at least a pivot nurse, a pharmacist, a medical oncologist, a nutritionist, and a social worker or psychologist [18]. The team’s size and diversity are determined according to the clients’ needs and the caseload of the cancer outpatient clinic. Cancer team processes should involve the following: initial assessment of patient needs from a holistic perspective; formal and regular interdisciplinary meetings to discuss complex patient-family biopsychosocial problems; development of coordinated interdisciplinary intervention plans; mastery of coordination procedures both upstream and downstream, supported by appropriate tools within the team and with external partners; and implementation of measures to ascertain the quality of the services offered. The overarching goal is to provide comprehensive cancer care and treatments to patients who come to the oncology clinics for investigative examinations, chemotherapy, or follow-up visits.

## Methods

### Study design and procedures

Our cross-sectional study used an *ex post facto* quasi-experimental design [19] to investigate potential associations by observing an existing condition (ITW in our case) and to evaluate the association between ITW intensity and the experience of care as reported by outpatients. According to Cohen et al., this design was deemed appropriate considering that ITW already occurred among cancer team members and that this variable is studied in retrospect for seeking the likely effects that the changes in this independent variable (High *vs.* Low ITW intensity) produce on a set of dependent variables (PREMs) [19].

### ITW intensity measure

To our knowledge, no validated tool was available to evaluate ITW intensity in oncology teams at the time we undertook the study [20, 21]. We developed an ITW intensity measure based on our structure-process-outcome conceptual framework. A list of potential qualitative indicators was then submitted to a group of nine experts including clinicians, managers, and researchers to test the content validity of our ITW intensity measure from both scientific and pragmatic points of view [22]. The resulting consensus led to a tool that included thirteen items grouped into five components (Fig. 1): 1) *Composition of the team and frequency of cancer care team meetings* (two items); 2) *Shared care philosophy among team members* (one item); 3) *Coordination mechanisms and tools* (three items); 4) *Leadership, responsibilities, and processes shared among clinicians and managers* (two items); 5) *Quality assessment activities* (five items). An assessment grid covering all of these dimensions was pre-tested in nine well-known clinics that had previously participated in our studies on cancer care transformation [23–26]. It was then administered by a member of the research team during an individual interview with the cancer clinic first-line manager. Structured response to items in the key informant interview was converted into quantifiable categories according to the seminal article by Caracelli and Green [27]. For each item (13), a score was created; the scores were then equally weighted and converted to a final ITW intensity score ranging from 0 to 10. Interview grid and quantification criteria are described in Additional file 1.

### Study settings and participants

The selection of study settings resulted from a stepwise strategy. An initial ITW score from all the outpatient oncology clinics in Quebec with a local or a regional mandate (*n* = 65) was established using the Ministry of Health and Social Services administrative database for monitoring the structure and processes of cancer care teams.

For feasibility reasons, clinics with very low patient volume were excluded. Clinics with the six highest and six lowest ITW scores were selected for a total of twelve clinics. Among these clinics, nine agreed to participate in the study, representing nearly 14% of all outpatient oncology clinics (*n* = 65) with a local or a regional mandate in Quebec (Table 1). Second, the data were collected from managers in each participating clinic using the ITW tool developed for the present study. Third, the outpatient clinics samples were divided into two categories: *Low ITW intensity* and *High ITW intensity*. Three clinics were classified as *Low ITW intensity* (scores ranging from 4.08 to 6.17), and six were classified as *High ITW intensity* (scores ranging from 7.88 to 9.75) (Table 1). A cut-off point was determined to maximize differences in ITW intensities when comparing participating sites. Greater score differences between ITW intensities were seen in relation to team process characteristics compared to team structure characteristics.

**Table 1:** Organizational characteristics of participating sites (*N* = 9)

Site	Mandate^a^	Geographic location	Academic-affiliation	Cancer team size^b^	ITW intensity	Participants^c^	
						N	(%)
A	Regional	Rural	Yes	Small	4.08	158	(11.5)
B	Regional	Urban	Yes	Large	4.71	202	(14.6)
C	Regional	Semi-rural	Yes	Large	6.17	158	(11.5)
D	Local	Rural	No	Small	7.88	98	(7.1)
E	Local	Rural	No	Small	8.13	86	(6.2)
F	Regional	Semi-rural	No	Large	8.38	140	(10.2)
G	Local	Rural	Yes	Small	9.13	143	(10.4)
H	Local	Urban	Yes	Small	9.50	214	(15.5)
I	Local	Urban	No	Large	9.75	180	(13.1)
Total						1379	(100.0)

^a^At the time of the study

^b^Large: cancer team with 8 or more professionals from various discipline; Small: fewer than 8 such professionals

^c^Participants with completed questionnaires included for the statistical analysis

Participants were recruited from the participating outpatient oncology clinics between October 2010 and November 2011. Minimal criteria were applied to maximize patient eligibility [28] and to increase the likelihood that participants would broadly represent cancer patients both seeking care in these outpatient clinics and having a minimal experience of care received from a team. Thus, patients were eligible if they were 18 years of age or over, had a confirmed cancer diagnosis (all cancers, all stages), had visited the oncology clinic at least once in the preceding twelve months, and could read and understand either French or English. All participants were recruited upon arrival at the oncology clinic whether for investigative examinations, chemotherapy, or follow-up visits.

Designated staff members were trained to identify participants who met our inclusion criteria and to provide them with information about the study in a standardized way. Patients interested in the study were given the following material: a cover letter describing the study and outlining the conditions if they agreed to participate; a self-administered survey questionnaire; and a stamped envelope to return the completed questionnaire by mail. Weekly contact with designated staff members and a reminder about the study was sent to patients two weeks after initial distribution of the questionnaire to improve response rate [29]. Participation in the survey was voluntary and anonymous.

### Patient-reported experience questionnaire

The questionnaire included thirty-three items grouped into six scales considered to measure the anticipated effects of ITW on the patients’ experience of cancer care. Four out of six cancer-adapted scales were derived from the French-language version of the Health System Responsiveness Questionnaire [30], validated by Tremblay et al. [26, 31]: *Prompt access to care* (four items; α = 0.77), *Person-centred response* (five items; α = 0.67), *Quality of patient-professional communication* (five items; α = 0.85), and *Quality of the care environment* (five items; α = 0.64). The *Continuity of care* scale (nine items; α = 0.77) was derived from a generic measure of continuity aiming to assess information gaps perceived by patients encountering several professionals [32]. The *Results of care* scale (five items; α = 0.82) was adapted from an instrument measuring perception of primary care services [33]. For all items, patients were asked to report their experience of care within the preceding twelve months using a four-point Likert scale (1 = never, 2 = sometimes, 3 = often, 4 = always). Scale scores were calculated as the average rating of all scale items. Thus, scores could vary between 1 and 4, with high scores indicating a more positive perception of cancer care.

Data were also collected on patient and organizational characteristics that could independently affect patient perception of the care experience [34, 35]. Patient socio-demographic characteristics were gender, age, education level, perceived financial situation, self-assessed health status, and perceived emotional distress (reverse-scored). Emotional distress was assessed using the six items from the Health Education Impact Questionnaire (heiQ) [36]. This scale is an adapted and validated French version for a cancer clientele [37, 38], measuring overall negative affective responses in relation to the impact of the illness. Variables related to self-reported patient clinical characteristics at time of recruitment were as follows: time since diagnosis, cancer site, treatment type, and presence of comorbidity.

Organizational characteristics were as follows: hospital mandate regarding oncology services (local or regional); university or non-university affiliated hospital; geographic location (urban, semi-rural, rural); and interdisciplinary team size and diversity (large = eight or more professionals from various disciplines; small = fewer than eight such professionals) [39, 40]. The patient questionnaire was available in French and English. The Quebec Ministry of Health and Social Care administrative database and oncology team managers were the main sources of information for organizational characteristics.

### Statistical analyses

Statistical analyses were performed using IBM SPSS Statistics 19.0 for Windows and SAS 9.2 for Windows. Descriptive statistics were computed for each questionnaire item and scale score. Since the distribution of PREM dimension scores were skewed toward higher values, these variables were dichotomized [41]. A dichotomous outcome variable was then created in which participants providing positive responses for more than 75% of a scale’s items (e.g., 3 out of 4 items: 4 out of 5 items) were classified as having a positive perceived experience (often, always), while the remainder were classified as having a less positive experience (never, sometimes) [41].

To test the association between ITW intensities and specific dimensions of patient-reported experience, each scale was considered separately as a dependent variable using logistic regression analysis. Because of correlated patient responses within sites, PROC GENMOD was used to fit logistic models. An odds ratio (OR) and a 95% confidence interval (CI) were calculated to measure the association between ITW intensities and each dependent variable. As per the Strobe Statement on observational studies [42], OR is the ratio of the odds of reporting a positive care experience among patients in high ITW intensity settings, compared to the corresponding figure among patients in low ITW intensity settings. The other patients, as well as organizational characteristics likely to affect patient-reported experience independently of ITW, were simultaneously entered into the multiple logistic regression to obtain an adjusted OR. Potential confounders [42] related to patient factors (gender, age, education level, perceived health status, emotional distress) and to organizational factors (mandate, geographic location, university affiliation, and cancer team size) were included in the final regression analysis.

## Results

### Response rate and baseline patient characteristics

Of the eligible patients (*n* = 1981) who were offered the questionnaire, 165 (9.0%) declined. Of the 1816 who agreed to receive the questionnaire, 1453 (80%) returned it by mail. A total of 1379 participants completed at least 80% of the items and were included in the analysis, for a response rate of 70%. The response rate in each site was similar and across high and low ITW intensity aggregates.

Table 2 presents patient sociodemographic and clinical characteristics. It also illustrates statistical differences between high and low ITW intensity aggregates. The mean age of the sample was 61, and the majority of participants were female. About 18% had completed primary school only. The most frequent cancer types were breast (26.5%), colorectal (21.4%), hematopoietic (15.9%), and bronchopulmonary (14.2%). Finally, 55.7% reported that they were consulting for a cancer diagnosed within the past year, and almost 90% said they had received chemotherapy in the past year, either alone or in combination with other treatments (surgery and/or radiotherapy).

**Table 2:** Patient sociodemographic and clinical characteristics (*N* = 1379)

Characteristics	Full sample		Low ITW		High ITW		Chi^2^ or *T*-test *p*-value
	Percent	n^a^	Percent	n^a^	Percent	n^a^	
Gender							
Female	61.9	845	63.8	305	60.9	540	0.288
Age (years)							
Mean age (SD)	61.0 (11.0)		61.3 (10.8)		61.0 (12.2)		0.597
18–49	15.7	214	13.6	65	16.8	149	0.076
50–69	61.5	839	65.5	313	59.3	526	
70–98	22.9	312	20.9	100	23.9	212	
Education level (completed)							
Primary	18.3	246	18.1	85	18.4	161	0.034
Secondary	44.4	598	45.7	215	43.8	383	
Business college/CEGEP^b^	15.7	211	18.5	87	14.2	124	
University	21.6	291	17.8	84	23.7	207	
Perceived financial status							
Financially comfortable	21.9	291	19.6	90	23.2	201	0.155
Earn enough	57.0	757	60.4	278	55.2	479	
Poor	18.8	249	18.5	85	18.9	164	
Very poor	2.3	31	1.5	7	2.8	24	
Cancer type							
Breast	26.5	359	29.1	138	25.0	221	<0.0001
Colorectal	21.4	290	23.2	110	20.4	180	
Hematopoietic	15.9	216	9.9	47	19.1	169	
Bronchopulmonary	14.2	192	17.5	83	12.3	109	
Female genital	4.6	62	5.7	27	4.0	35	
Other	17.5	238	14.6	69	19.1	169	
Time since diagnosis (years)							
< 1	55.7	759	57.3	271	54.8	488	0.003
1 to 3	27.7	377	30.7	145	26.1	232	
≥ 3	16.7	227	12.1	57	19.1	170	
Treatment type							
Chemotherapy only	39.0	519	36.6	173	40.3	346	<0.0001
Chemotherapy + other treatment	49.0	653	55.8	264	45.3	389	
Other	6.8	91	5.7	27	7.5	64	
None	5.1	68	1.9	9	6.9	59	
Health status (self-assessed)							
Good	50.4	683	50.1	237	50.6	446	0.871
Poor	49.6	672	49.9	236	49.4	436	
Comorbidities (self-reported)							
0	34.3	473	33.7	162	34.6	311	0.891
1 to 3	59.4	819	59.7	287	59.2	532	
More than 3	6.3	87	6.7	32	6.1	55	
Emotional distress^c^							
Low	47.7	647	51.1	241	46.0	406	0.075
High	52.3	708	48.9	231	54.0	477	

^a^n may vary per characteristic due to missing value

^b^In Quebec, business colleges and CEGEPs are post-secondary institutions providing pre-university education (2 years) or specialized vocational programs (3 years)

^c^Form heiQ emotional distress score (6 items), Low: lower than mean (normal distribution); High: higher than mean

### Patients’ overall perception of their care experience

In general, patients reported positive perceptions of the various aspects of their care experience, with scores ranging from 3.34 (SD = 0.69) to 3.75 (SD = 0.01) out of 4 for all scales (Table 3). The proportion of patients reporting a positive perception of their experience was also high for most scales (ranging from 64.6 to 75.5%) except for *Prompt access to care* (45.3%) (Table 4).

**Table 3:** Description of the six dimensions of patient-reported experience

Dimension	Mean score^a^	SD^b^
Prompt access to care	3.34	0.69
Person-centred response	3.66	0.41
Quality of patient-professional communication	3.65	0.56
Quality of the care environment	3.72	0.37
Continuity of care	3.75	0.01
Results of care	3.60	0.55

^a^Theoretical score range: 1 to 4; higher scores indicate more positive perception of experience

^b^SD: Standard deviation

**Table 4:** Association between interdisciplinary teamwork intensity and the dimensions of patient-reported experience measures (PREMs)^a^

Dimensions of patient-reported experience	Positive PREMs (Overall %)	Adjusted^c^ OR^b^ (95% CI)	*p* value
Prompt access to care	(45.3)	3.99 (1.89–8.41)	0.0002
Quality of patient-professional communication	(64.6)	2.37 (1.25–4.51)	0.0325
Continuity of care	(75.5)	2.18 (1.07–4.45)	0.0324
Person-centred response	(73.4)	2.11 (1.05–4.24)	0.0377
Results of care	(64.9)	1.31 (0.68–2.52)	0.4192
Quality of the care environment	(75.3)	0.66 (0.31–1.39)	0.2740

^a^Low ITW intensity is the reference

^b^OR: odds ratio

^c^Adjusted for patient characteristics (health status, age, gender, education level, emotional distress) and organizational characteristics

Associations between high ITW intensity and dimensions of care are reported in Table 4, which shows adjusted OR. Only *Prompt access to care* was positively associated with high ITW intensity when unadjusted OR was calculated. After adjusting the multiple regression model for potential confounders related to patient and organizational factors, four of the six dimensions of care showed significant positive associations characterized by high ITW intensity. The strongest association was found with *Prompt access to care* (OR = 3.99; CI = 1.89–8.41). *Quality of patient-professional communication* was more than two times positively associated with high ITW intensity (OR = 2.37; CI = 1.25–4.51), followed by *Continuity of care* (OR = 2.18; IC = 1.07–4.45) and *Person-centred response* (OR = 2.11; IC = 1.05–4.24). No significant association was found between ITW intensity and *Results of care* (OR = 1.31; CI = 0.68–2.52) or *Quality of the care environment* (OR = 0.66; CI = 0.31–1.39).

## Discussion

This study aimed to compare ITW intensity with PREM dimensions of care among cancer outpatients. The results show that cancer patients treated in outpatient clinics characterized by high ITW intensity are more likely to report positive perceptions regarding four PREMs dimensions (*Prompt access to care*, *Person-centred response*, *Quality of patient-professional communication*, *and Continuity of care*), compared to patients treated in clinics with low ITW intensity. The results thus support the hypothesis whereby higher ITW intensity within cancer care teams translates into a more positive patient perception of their care experience.

### ITW intensity variation

The current literature on cancer teams focuses on their objectives and their organization [6] or on team effectiveness [5], and it does not integrate teamwork components into proper structure-process-outcome associations. Our ITW tool administered through interview with front-line manager provided original data not only on team structure (Composition of the team, Frequency of cancer care team meetings) but also on critical aspects of the teaming processes (Shared care philosophy among team members, Coordination mechanisms and tools, Leadership, Responsibilities and processes shared among clinicians and managers, Quality assessment activities). Those results are consistent with the work of Salas et al. who developed a comprehensive model for enhancing teamwork skills in healthcare (i.e., The Big Five in Teamwork [43]). Although the ITW tool is in its infancy, it allowed revealing variation in ITW intensity and its association with cancer patient experience.

### Patient-reported experience

To the best of our knowledge, and considering reviews in the field of interdisciplinary teamwork and patient-reported outcomes, our study is one of the few to demonstrate the extent to which positive patient perception of *Prompt access to care* is associated with high ITW intensity within care teams [5–7]. Positive patient perception of *Prompt access to care* is an important anticipated effect of high ITW intensity. Indeed, access to cancer care from first symptoms to treatment is a topic of concern for researchers and policy makers [44–47]. In our study, *Prompt access to care* was defined as patients’ perception of their ability to reach or see an oncology professional, as required, at various times of the day and on different days of the week. Outpatients, who are by definition outside the hospital setting, must employ a number of self-management strategies to cope with their cancer symptoms and treatment. When these coping strategies fail, the patients’ unmet needs may require prompt access to care professionals [48]. A possible explanation of the association between high ITW intensity and positive perception of *Prompt access to care* is that ITW is aimed at improving care coordination by facilitating appropriate referral mechanisms to the relevant team member [49]. If this explanation is correct, our results would suggest that high ITW intensity has the potential to break down the “silos” in the health care system, reduce the barriers between multiple health professionals located in different settings (e.g., ambulatory oncology clinic, hospital, home care), and blur the arbitrary distinction between medical and psychosocial needs. Indeed, patients’ positive perception of *Prompt access to care* is an important positive effect of high ITW intensity.

Positive perception of *Quality of patient-professional communication* was the second most positive effect of high ITW intensity documented in this study. Such communication is considered vital to quality patient care, particularly in oncology settings [50, 51]. Patient-professional communication reflects the ability of a professional to listen to the patient, provide a clear response, and embrace a shared decision-making (SDM) approach. SDM is directly related to patient-professional dynamics. In the oncology field, high levels of satisfaction and confidence in treatment decisions are positively associated with SDM and are related to low levels of patient depression. These associations are independent of the patients’ preferred level of participation (passive or active) in SDM [52]. On the one hand, patients report their symptoms and self-management activities to a team member who, in turn, provides additional information about treatment outcomes and shares the information with other team members. This information is valuable because it enables team members to tailor interventions to the individual by taking into account his or her specific characteristics and behavior patterns. On the other hand, it is well documented that poorly managed communication in the oncology field can result in unnecessary treatment and emotional distress for patients, thus negatively affecting their care experience [51, 53, 54]. This may explain why lower ITW intensity was less strongly associated with positive perception of *Quality of patient-professional communication* in our study.

The association between positive perception of *Person-centred response* and high ITW intensity in the context of cancer care was another important finding. Since the Institute of Medicine’s seminal publication [55], the patient-centred approach has been considered the hallmark of high-quality care. Over the past two decades, patient-centred care has become internationally recognized as a dimension of the broader concept of high-quality health care, and many countries are now designing and implementing strategies and programs in this regard [56]. In a recent systematic review on cancer team effectiveness, only one study out of eleven addressed patient-centred care as an outcome indicator during active treatment. A qualitative multiple-case study completed with two interdisciplinary cancer teams from a Canadian teaching hospital reported that integrating patient values and preferences was still difficult for cancer care professionals, and patients were often expected to follow the rules established by professionals [57]. Bilodeau et al. concluded that two conflicting models shape the organization of oncology services: patient-centred discourse and professional-centred practice [57]. The association between high ITW intensity and positive perception of person-centred response may bring us to envision ITW as a way of accommodating the strengths of these two models, cancer care being seen as a professional service as well as a human relationship between patients and health professionals.

Our results indicate that patients receiving care in outpatient clinics with high ITW intensity are twice as likely to have positive perceptions of *Continuity of care*, compared to patients in clinics with low ITW intensity. Indeed, continuity of care is not simply about seeing the same health care professional at every visit; it is about perceiving that the interdisciplinary team uses all the information at its disposal (clinical and personal) for effective care planning. Health care professionals in teams with high ITW intensity tend to share the professional responsibility of oncology treatment and follow-up, offering more holistic patient care and allowing more opportunities to diagnose cancer recurrence. High ITW intensity involves paying attention to and anticipating the potential impact of cancer and its treatment, dealing with service silos, managing consequences for the whole person, and ensuring that important aspects of care have not been overlooked by the health professionals involved [58]. When professionals work together, attention can be directed to overcoming barriers facing patients, such as lack of knowledge of symptoms; late diagnosis and treatment due to fear; anxiety about disruption of work; child care problems; financial concerns; and unreliable transportation [59, 60].

Contrary to the conclusions of systematic reviews on ITW [5–7], we did not find an association between high ITW intensity and positive patient perception of *Results of care*. One possible explanation may be that the association between ITW intensity and perceptions of *Results of care* has multiple determinants, creating an indirect association. Other types of interventions, such as tumor boards focusing on therapeutic regimens, systematic symptom evaluations, and interventions by individual professionals, may act on the pathway between ITW intensity and patient-reported experience.

There was no association between high ITW intensity and positive perception of *Quality of the care environment*. Perhaps patients perceive the patient-professional-team relationship as having more importance than *Quality of the care environment*. Another explanation may be that basic amenities and professional courtesy are not directly related to ITW intensity. Some studies suggest that the responsibility to create a healing environment belongs all those working in a cancer setting, regardless of ITW intensity, and that this responsibility is embedded in complex relationships between professional practices, setting, and care providing processes [61].

### Strengths and limitations of the study

A conceptual framework was used to ensure methodological transparency. Our measurements of interdisciplinary teamwork focused on both team structure and process items that characterize teams with high or low ITW intensity. We used the available validated instruments, which were cancer-adapted and had good reliability scales overall. No validated tool was available to evaluate ITW intensity in teams such as those in Quebec that include a diversity of professionals [4, 20, 21]. The general view in the literature is that the traditional criteria for scientific validity (e.g., internal consistency) do not by themselves guarantee usefulness to practitioners. Considering our pragmatic stance with the ITW tool, our work concentrated on content and pragmatic validity [62]. Pragmatic validity of knowledge can be judged by the extent to which intended consequences can be achieved by using particular instruments.

We thus carefully developed a measurement tool specific to oncology care. Although the tool is still under development, we used recognized procedures to ensure content validity evaluation [63]. The tool could be used in its current form to measure ITW intensities of other cancer care teams that are similar in size and include a diversity of professionals focusing on comprehensive patient-centred cancer care. However, it should not be used for interdisciplinary teams that are solely oriented toward medical treatments (e.g., cancer conferences or tumor boards). Potential misclassification of ITW intensity was mitigated by using information from the administrative database of the Ministry of Health and Social Services and interviews with front-line managers from each participating clinic [64].

Certain limitations may also be related to the PREMs scale. The tool used to measure perception of *Results of care* and *Quality of the care environment* was derived from the primary care sector. It is possible that it was not sufficiently sensitive to fully capture the effects of high ITW intensity, since all cancer teams work together to some extent. Finally, reliability (alpha = 0.64) of the *Quality of the care environment* scale (alpha = 0.64) was the lowest of all the scales used in the study, despite the fact that it was a cancer-adapted validated scale [26]. This may have reduced the ability to identify significant associations. Overall, it is difficult to compare our results with previous studies in the oncology setting [5, 40, 65] due to significant differences in study design, the conceptualization and measurement of interdisciplinary teamwork, and the dimensions of patient-reported differences. Nevertheless, our study contributes to research aiming to provide evidence for the effects of ITW.

Whereas *ex post facto* quasi-experimental design is not optimal [19], it is important to realize that no other study design was possible, since ITW is already implemented to varying degrees in all cancer teams in Quebec [66]. Given the high response rate among patients, the diversity of organizational characteristics of the clinics that were included, and our careful assessment of ITW intensity, the results of this study could be generalized to patients treated and followed up in similar settings [67]. Finally, considering the characteristics of our sample, and because access to medical services is universal in Quebec, we feel cautious about generalizing our results to other health care systems. Nevertheless, our study has strong internal validity due to the robustness of its methodology.

## Conclusions

Our quasi-experimental study makes an original contribution by demonstrating the association between interdisciplinary teamwork and specific critical aspects of the cancer care experience after adjusting for potential individual and organizational confounders. It represents an effort to produce evidence demonstrating the value and impact of team structure and teamwork processes in the context of cancer care. It also provides an original contribution to the measurement of interdisciplinary teamwork outcomes. Our study refines the portrait of what works in team-based care within the broader context of health care transformation. This is an important contribution considering that teamwork is a key concept promoted in most national cancer programs worldwide. Knowledge about the transformative capacity of teamwork requires further research to investigate more closely how teamwork contributes to a more positive experience, for whom and under what conditions. A better understanding of the association between teamwork and patient self-reported experience would have a positive impact on decisions related to the modernization of cancer services and help to better respond to the needs of cancer patients interacting with the health care system.

## Additional file

Additional file 1: ITW tool components and Patient-Reported Experience Measures (PREMs) questionnaire items. Description: **Table S1.** presents the interview grid items to determine ITW intensity, criteria and indicator for quantification of qualitative data. In reference to the PREMs questionnaire used in the study, questions in **Table S2.** were designed to measure the cancer experience from the patient’s perspective; questions in **Table S3.** were designed to document the sociodemographic, clinical, and organizational characteristics that were potential confounders. (PDF 106 kb)

## Abbreviations

ITW: Interdisciplinary teamwork
OR: Odds ratio
PREMs: Patient-reported experience measures
SCP: Survivorship care plan
SD: Standard deviation
SDM: Shared decision-making
