# Supplementary material for: Effects of interdisciplinary teamwork on patient-reported experience of cancer care
Source: BMC Health Serv Res. 2017 Mar 20;17:218. doi: 10.1186/s12913-017-2166-7 (PMC5360056; doi:10.1186/s12913-017-2166-7)
Supplement: Additional file 1: — ITW tool components and Patient-Reported Experience Measures (PREMs) questionnaire items. Description: Table S1. presents the interview grid items to determine ITW intensity, criteria and indicator for quantification of qualitative data. In reference to the PREMs questionnaire used in the study, questions in Table S2. were designed to measure the cancer experience from the patient’s perspective; questions in Table S3. were designed to document the sociodemographic, clinical, and organizational characteristics that were potential confounders. (PDF 106 kb) [file 12913_2017_2166_MOESM1_ESM.pdf]

Additional File 1: ITW tool components and Patient-Reported Experience Measures questionnaire items

Table S1 ITW tool components

**Dimension and items**	**Criteria**	**Quantification[a]**
Team structure		
**Composition of the team[b]**	• ≥ 6 members / 8 = 3; < 6 members / 8 = 0	0 to 3
**Team meeting**		0 to 3
• Formal meeting schedule	• Yes = 1,5; No = 0	
• Frequency	• ≤ monthly = 1.5; > 1 monthly = 0	
Team processes		
**Shared philosophy among team members**	• Seeking team tasks to be performed<br>Yes = 1; No = 0	0 to 1
**Coordination mechanisms and tools**		0 to 3
• Clear professional roles with reference criteria	• Access to written referral criteria to various team members<br>Yes = 1; No = 0	
• Patient's needs assessment tools	• Ongoing utilization of assessment tools<br>Yes = 1; No = 0	
• Interdisciplinary care plan	• Written and shared care plans<br>Yes = 1; No = 0	
**Leadership, responsibilities, and processes shared among clinicians and managers**	• Identified oncology-related medical leader<br>Yes = 1; No = 0<br>• Identified first-line manager with clinical expertise in cancer care<br>Yes = 1; No = 0	0 to 2
**Quality assessment activities**		0 to 10
• Continuing education	• Regular activity program = 2<br>Occasionally = 1; None = 0	
• Backup behaviors (support between teams members)	• Planned co-development and debriefing = 2; Occasionally = 1; None = 0	
• Identified quality manager	• Yes = 2; No = 0	
• Regular patient satisfaction survey	• Yes = 2; No = 0	
• Team effectiveness evaluation	• Regular activity program = 2;<br>Occasionally = 1; None = 0	

[a] Transformation for a total of 0 to 10 ITW intensity score
[b] According to Cancer directorate, Ministry of Health

Table S2 Six dimensions of the Patient-Reported Experience Measures questionnaire items

Item	*Question: In the past 12 months…*
**Prompt access to care**	
1	On weekdays it was possible to contact a professional at the cancer clinic by phone when needed
2	On weekdays it was possible to consult the cancer clinic on the same day in cases of emergency or complications
3	During evenings, at night and on weekends it was possible to contact a hospital professional by phone in cases of emergency or complications
4	The amount of time spent in the waiting room before a consultation was reasonable
**Person-centred response**	
5	Professionals provided you with the information you needed within a reasonable time given your health status
6	Professionals asked for your consent before beginning a treatment or test
7	Professionals addressed all your needs
8	The confidentiality of your personal information was respected
9	Your close relatives were encouraged to be present as much as you wanted
**Quality of patient-professional communication**	
10	Professionals listened to you attentively
11	Professionals gave you simple explanations that were easy to understand
12	Professionals provided you with enough time to ask all the questions you had about your illness and treatments
13	Professionals encouraged you to participate as much as you wanted in decisions regarding your care, treatment and tests
14	Professionals helped you assess the "pros" and "cons" regarding care, treatment and tests
**Quality of care environment**	
15	Professionals treated you with respect
16	The waiting room was comfortable
17	The administrative staff treated you respectfully
18	Tests and treatments were conducted in a way that respected your physical privacy
19	You were able to speak privately with the professionals at the clinic
**Continuity of care**	
20	The information about your cancer and treatments was sent to your family doctor

Item	*Question: In the past 12 months…*
21	You were informed of the different steps in your treatment and care
22	Someone helped you obtain the care you needed
23	At least one professional was well informed about your situation
24	The professionals gave you contradictory information
25	One of the professionals consulted was not aware of your recent medical history
26	You had to redo tests
27	A professional you consulted was not aware of the treatment changes recommended by another professional
28	You had to repeat information that should have been in your medical file
**Results of care**	
29	Professionals helped you control your pain and symptoms
30	Professionals helped you manage your emotions and stress regarding your illness
31	Professionals motivated you to adopt or maintain good lifestyle habits such as eating healthy, exercising and quitting smoking
32	Professionals encouraged you to follow your treatments
33	Professionals met your overall needs

Table S3 Questionnaire items measuring sociodemographic, clinical, and organizational characteristics

Item	*Variable*	*Operationalization*
**Patients' sociodemographic characteristics**		
1	Age	18-49 years; 50-69 years; 70 years and older
2	Sex	Male; Female
3	Education level (completed)	Primary ; Secondary; Business college/CEGEP**; University
4	Self-assessed health status	Poor; Good
5	Emotional distress*	Low; High
**Patients' clinical characteristics**		
6	Time since diagnosis	< 1 year; 1-3 years; ≥ 3 years
7	Cancer type	Colorectal, female genital, prostate, hematopoietic, breast, head and neck, bronchopulmonary, other
8	Treatment type (in the past 12 month)	Chemotherapy; radiotherapy; surgery; other; none
19	Comorbidities	No comorbidity; 1-3 comorbidities; more than 3 comorbidities
**Organizational characteristics**		
10	Mandate	Local mandate; regional mandate
11	Academic affiliation	University hospital; community hospital
12	Geographic location	Urban; semi-rural; rural
13	Cancer team size and diversity	Cancer team with 8 professionals from different disciplines and more = large; fewer than 8 = small)

* Emotional distress (6 items)

** In Quebec, Business college/CEGEP are post-secondary institutions that provide pre-university education (2 years) or specialized vocational programs (3 years)
